# Neural activity in macaque medial frontal cortex represents others’ choices

**DOI:** 10.1038/s41598-017-12822-5

**Published:** 2017-10-04

**Authors:** Rossella Falcone, Rossella Cirillo, Stefano Ferraina, Aldo Genovesio

**Affiliations:** Department of Physiology and Pharmacology, Sapienza - University of Rome, Piazzale Aldo Moro 5, 00185 Rome, Italy

## Abstract

Predicting the behavior of others is a fundamental skill in primate social life. We tested the role of medial frontal cortex in the prediction of other agents’ behavior in two male macaques, using a monkey-human interactive task in which their actor-observer roles were intermixed. In every trial, the observer monitored the actor’s choice to reject it for a different one when he became the actor on the subsequent trial. In the delay period preceding the action, we identified neurons modulated by the agent’s identity, as well as a group of neurons encoding the agent’s future choice, some of which were neurons that showed differential patterns of activity between agents. The ability of these neurons to flexibly move from ‘self-oriented’ to ‘other-oriented’ representations could correspond to the “other side of the coin” of the simulative mirroring activity. Neurons that changed coding scheme, together with neurons exclusively involved in the prediction of the other agent’s choice, show a neural substrate for predicting or anticipating others’ choices beyond simulation.

## Introduction

Social relationships are an integral part of everyday life among primates. A basic feature of social interaction is the ability to predict the behavior of other individuals. Although rhesus monkeys fail to understand and predict others’ beliefs^1^, they are able to predict some aspects of others’ behavior. Monkeys are also able to monitor the behavioral outcome of another monkey’s actions^2,3^ and to learn from other individuals^4–7^.

Until a few years ago, accumulating evidence from single neuronal recording studies in monkeys has suggested the importance of the fronto-parietal mirror system in understanding the actions of others in monkeys^8–10^, and later in humans^11,12^. The activity of these neurons has been interpreted as a “simulation” of other agent’s actions when they belong to the observer’s repertoire, like grasping to eat or place food in a container^13^.

Only more recently has the research focus extended to the importance of self vs. others’ action and reward differentiation, as well as monitoring^14^, and neurons that respond to other’s actions separately from one’s own actions in both pre-supplementary motor area (pre-SMA) and supplementary motor area (SMA) of monkeys^2^ and in human SMA^15^ have been found.

It remains unknown whether, in addition to action representation, these areas carry out predictive or anticipatory signals of others’ actions. To address this question, we used an interactive paradigm in which a delay period was introduced before the partner’s action.

Our aim was to explore the neural correlates of representing the others’ future actions using a task in which the other agent’s future behavior could be represented without complex reasoning about his intentions or beliefs. We asked whether the same neurons involved in planning one’s own actions were also involved in representing the future actions of others by a simulation process, or whether it depended on the activation of a separate neural network.

We examined the role of areas 8 and 9 of the posterior part of the medial prefrontal cortex (pmPFC), at the level of single neurons, in the representation of others’ future behavior.

The few studies performed in pmPFC of monkeys have indicated its involvement in the selection of appropriate strategies^16–18^ and in the control of eye and ear movements^19^. For a complete comparison with the previously observed activity^2^, we extended the study additionally to the pre-SMA and SMA areas.

## Results

### Behavior

**Figure 1:**
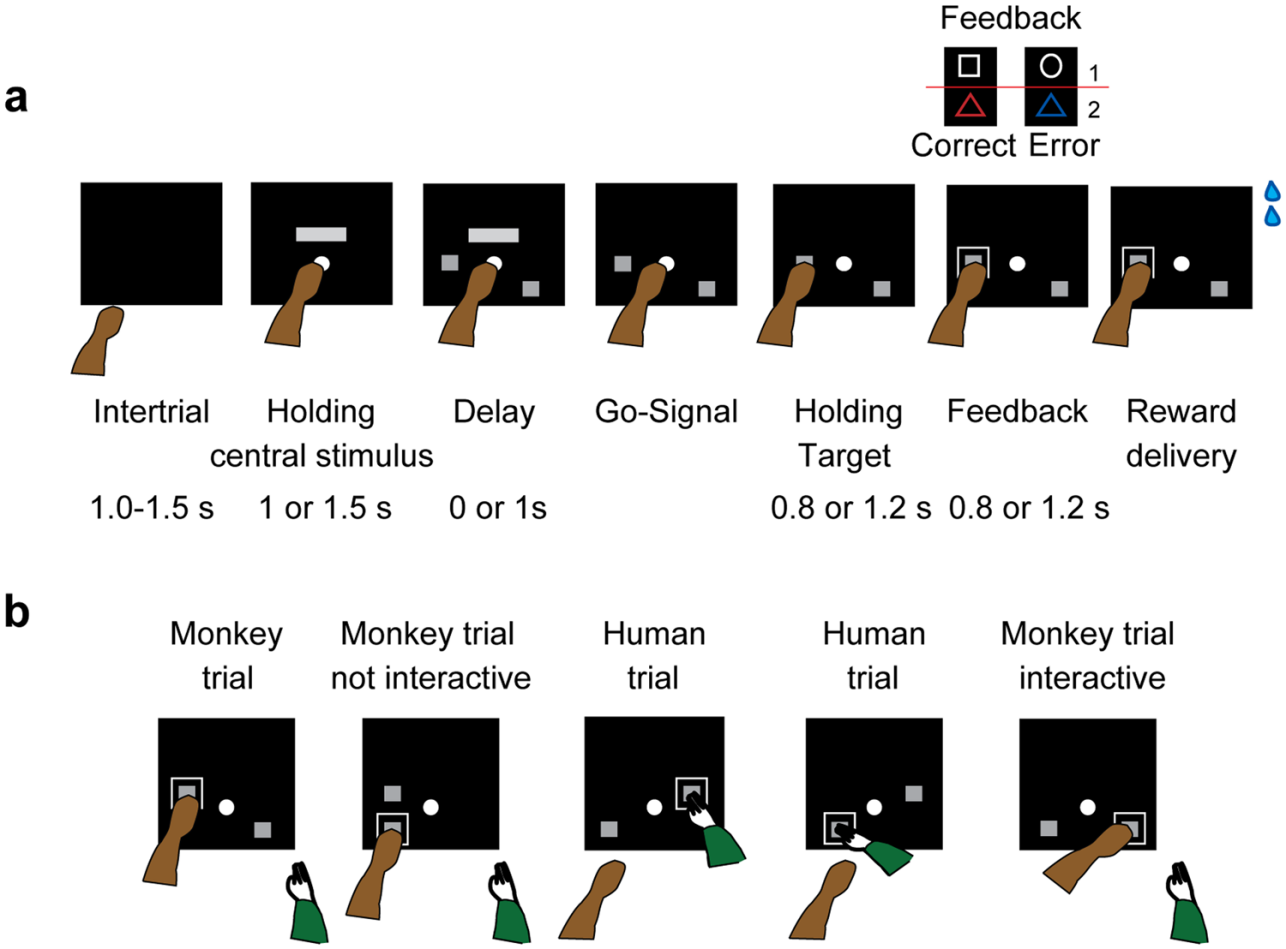
Task design. (**a**) Sequence of task events. Each black rectangle represents the video screen. Above are displayed the four visual stimuli used in a pair as either positive or negative feedbacks (pairs 1–2). Each grey rectangle on the video screen represents a positional target. Two targets are shown, one at center left and the other at the bottom right. (**b**) Example of choices in trials in which human and monkey switch actor and observer roles. Monkey’s trials after a human trial are designated as *interactive* trials; those after a monkey’s trial are designated as *not interactive*. The first trial of each session cannot be classified, since there is no preceding trial. The green arm represents the human.

Two monkeys performed the Nonmatch-to-goal task (NMTG; see Materials and Methods and Fig. 1a for detailed description of the sequence of events) while they were interacting with a human partner. The trial started when a central stimulus at the center of the screen was touched and a horizontal bar appeared above it. After a time, continuing to hold the central stimulus, two spatial targets appeared in two of four possible screen positions. After a delay, the horizontal bar disappeared serving as a “go” signal and triggering the response. Next, a visual feedback was presented, followed by a reward in correct trials.

Monkey and human reversed their role as actor and observer (see Materials and Methods and Fig. 1b for detailed description of the interaction with human partner). The human turn was not cued, but determined by the human partner, who decided when to initiate a trial by moving his arm toward the screen during an intertrial period, ready to touch the central stimulus when it appeared on the screen. After a trial correctly executed by a human, monkeys received reward as in the trials correctly executed by themselves.

The performance of the human partner was 99% correct responses with both monkeys. The proportion of human trials across all sessions was 39% (SEM: ±0.60) in monkey 1 and 36% (SEM: ±0.79) in monkey 2.

The trials were based on a Nonmatch-to-position rule, where the actor (the monkey or the human) had to reject the target chosen in the previous trial, which always reappeared in a new trial, and choose the alternative target presented in one of the other three possible positions (an example sequence of trials is shown in Fig. 1b). The human partner performed 1, 2, or 3 consecutive trials, and rarely 4 (27.9%, 45.8%, 25.8% and 0.4% with monkey 1; 33%, 44.3%, 22.5% and 0.2% with monkey 2, respectively).

In most cases, the human partner started his sequence of trials after 2, and up to 6 (M ± SD: 4 ± 1.7), completed monkey trials (correct or error).

We distinguished the monkey’s trials as *interactive* and *not interactive* based on whether the previous trial was performed by the human or by the monkey, respectively (Fig. 1b). Both monkeys performed the task accurately; average performance in *not interactive* and *interactive* trials was, respectively, 97% and 91% correct responses in monkey 1 (SEM: ±0.50 and 1.40), 98% and 95% in monkey 2 (SEM: ±0.32 and 0.76). For monkey 1, the mean RT in the *not interactive* condition was lower than the mean RT in the *interactive* condition (M ± SEM: 330.4 ms ± 11.9 and 375 ms ± 20.05), *t*(88) = −1.91, *p* = 0.03 (one- tailed). No significant difference was found for monkey 2 (M ± SEM: 279.8 ± 8.14 and 274.5 ± 6.54), *t*(148) = 0.51, *p* = 0.69, (one-tailed).

We examined the eye movements of monkey 1 during correct trials of 1 s delay, preceded by a correct trial. We examined only the trials in which the two targets (one to choose and the other to discard) were presented pseudorandomly one to the right and the other to the left side of the screen. Monkey 1 looked more frequently at the target to choose, after targets onset in both monkey and human trials. Overall, we observed an increase in the proportion of trials in which the monkey was looking at the target to choose at the end of the delay in monkey trials and slightly later in the human trials, after the human movement onset (Supplementary Fig. S1). After target selection, we observed that the monkey looked at the chosen target more than the unchosen one, for both monkey and human trials. The eye movements of monkey 2, however, could not be examined due to technical problems during the eye recordings.

### Single neuron activity

While monkeys were performing the interactive NMTG task, we recorded non-simultaneous activity from three areas of medial frontal cortex: pmPFC, SMA and pre-SMA. The neural population included 273 total cells (87 for monkey 1 from 35/45 sessions and 186 for monkey 2 from 64/75 sessions), all recorded for at least 20 correct monkey trials and 20 correct human trials.

We recorded 64 cells in SMA (15 from M1, 49 from M2), 81 cells in pre-SMA (27 from M1, 54 from M2), and 128 cells in pmPFC (45 from M1, 83 from M2).

We focused the analyses on the delay period before the go signal because this time window contains the information relevant for (i) target choice in monkey trials, and (ii) predicting the future choice before the action in human trials.

**Figure 2:**
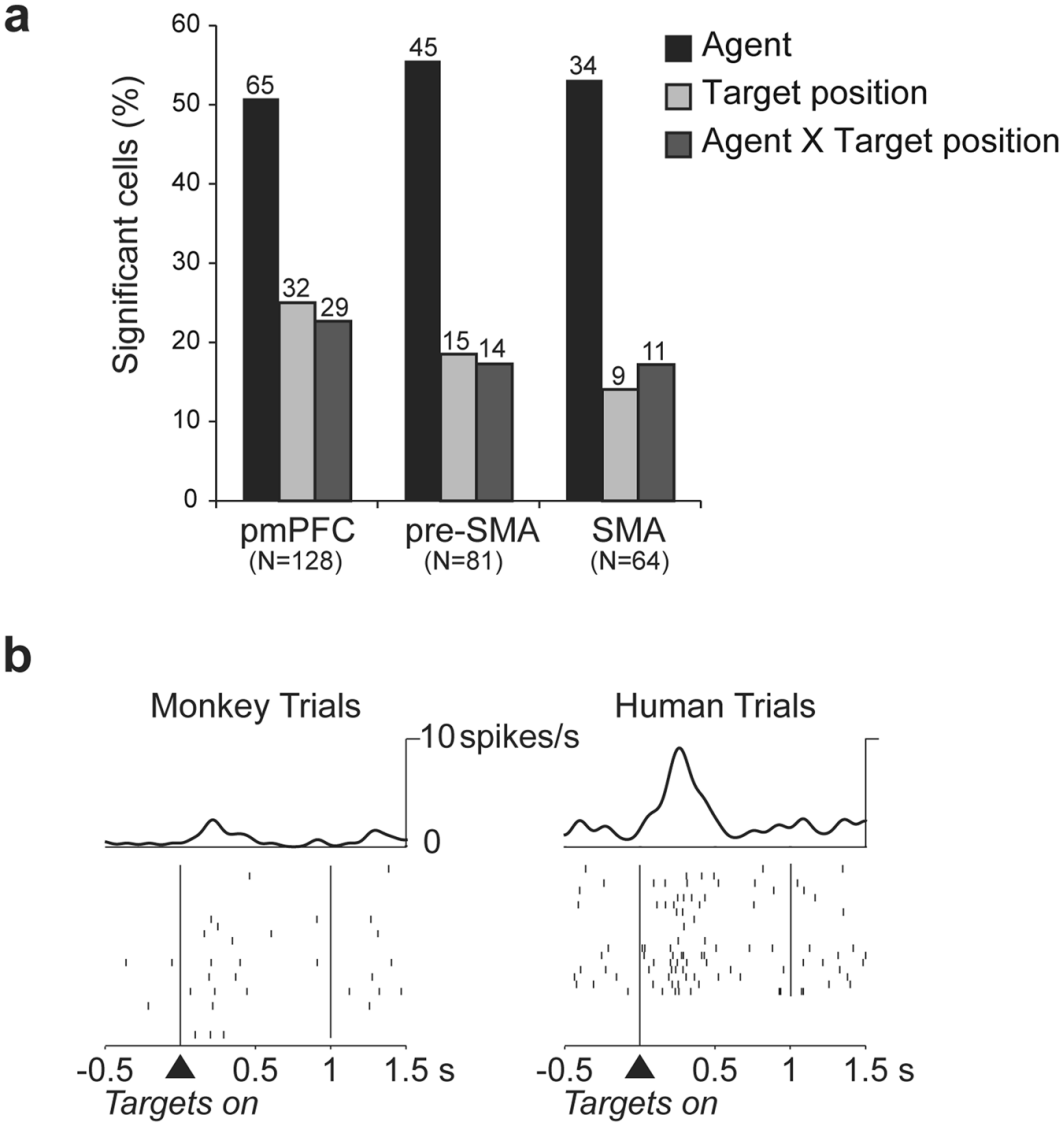
Neurophysiological results and a cell modulated by the agent. (**a**) Percentages of cells with a significant effect of agent, target position and their interaction during the delay period for each of the three brain areas: pmPFC, pre-SMA and SMA. (**b**) Example cell modulated by the agent performing the trial. The cell has higher activity in the first part of the delay period in the human trials compared to monkey trials. The trials are sorted by agent (irrespective of target position).

In all three areas from which we recorded, almost half of the cells were modulated by the identity of the actor (human or monkey), as indicated by two-way ANOVA (factors: agent and target position). A smaller proportion of cells represented the target position, again with comparable distributions between areas (Fig. 2a). Figure 2b shows an example of a neuron with higher activity in the trials performed by the human agent in the delay period.

**Figure 3:**
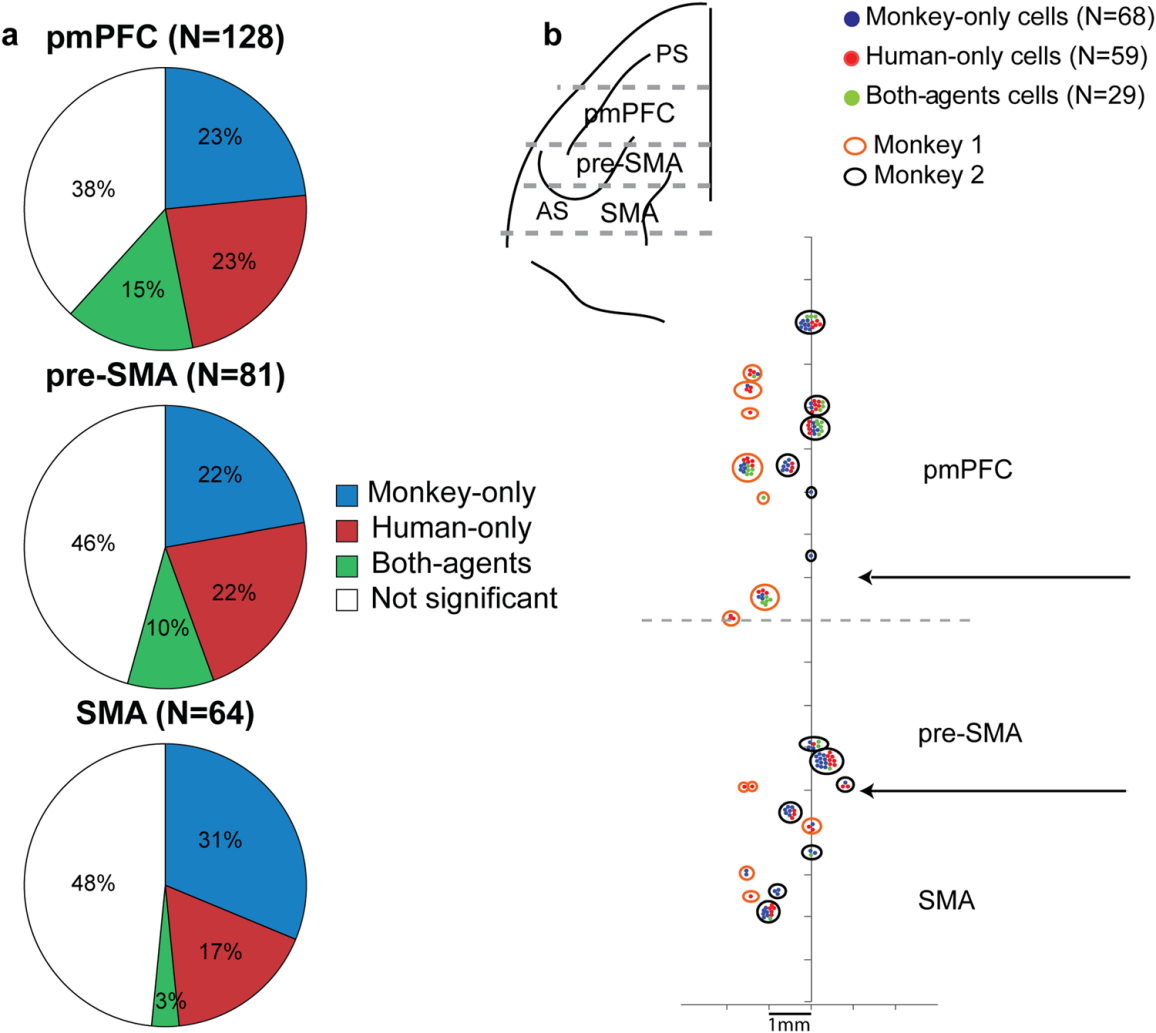
Percentage and distribution of cells among the recording areas. (**a**) Percentages of cells with a different response according to the target position only in monkey trials (monkey-only cells), only in human trials (human-only cells), or in both monkey and human trials (both-agents cells) and with no different response according to the target position (not significant cells) for each of the three brain areas of the medial frontal cortex (pmPFC, pre-SMA and SMA). (**b**) Top left – top view of the left frontal lobe derived from MRI of monkey 2. Dashed lines represent the estimated boundaries in the anteroposterior axis of the three brain areas: pmPFC, pre-SMA, and SMA. PS, principal sulcus; AS, arcuate sulcus. Bottom right - distribution of monkey-only, human-only and both-agents cells among the three brain areas of the medial frontal cortex. Each dot represents the cells classified in the three main groups of monkey-only (blue), human-only (red) and both-agents (green) cells from monkey 1 (orange circles) and monkey 2 (black circles). The two horizontal arrows indicate the estimated borders between the SMA and pre-SMA, and pre-SMA and pmPFC. The vertical line represents the cortical midline.

Based on *post-hoc* analysis on the total cell population (see Methods), we defined the cells as: monkey-only, human-only, and both-agents cells (Fig. 3a). The monkey-only cells and the human-only cells showed spatial target selectivity only in monkey or only in human trials. In contrast, the both-agents cells presented spatial target selectivity in both human and monkey trials. The cells with no significant positional differences either in human or in monkey trials did not belong to any of these three categories.

**Figure 4:**
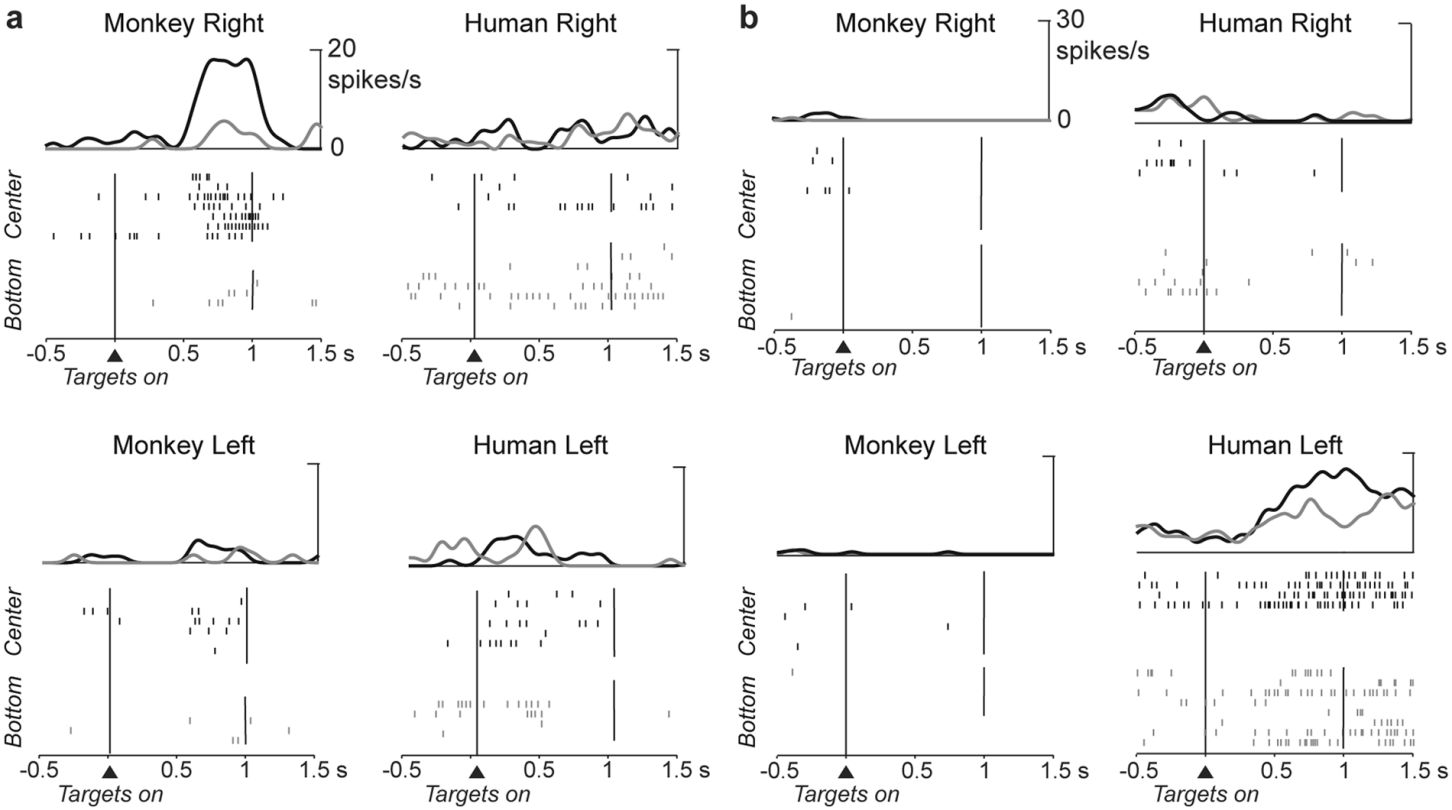
Spike raster plot of a monkey-only cell and a human-only cell. (**a**) Monkey-only cell. Cell showing a preference for the center right target position in monkey trials during the delay period. The same cell is not selective for the target position in the human trials. (**b**) Human-only cell. Cell with a preference for center and bottom left target positions in the human trials. This cell shows very low activity in the monkey-only trials, with no spatial selectivity. Each segment in the raster plot indicates the cell discharge relative to the targets onset with spike density averages above each raster.

The proportion of monkey-only and human-only cells was similar in all the three areas examined (monkey-only: χ^2^ (2, *N* = 273) = 1.84, *p* = 0.40; human-only: χ^2^ (2, *N* = 273) = 1.0, *p* = 0.60), indicating that in each recording area of the medial frontal cortex there was a representation of the human agent’s future choices and of monkey’s future choices. The proportion of both-agents cells was significantly different between the three areas (χ^2^ (2, *N* = 273) = 6.24, *p* = 0.04). We found that neurons belonging to different categories were interspersed, indicating no topographic segregation within each area (Fig. 3b). Figure 4a shows an example of a monkey-only cell with preference for the center right target. Figure 4b shows a human-only cell with predictive activity for left targets, but without any spatial selectivity in the delay period of monkey trials.

We also explored whether human-only and monkey-only cells could be identified simultaneously within the same recording session. We found that on 39 sessions with at least one human-only cell identified, and with at least 2 recorded cells, 18 of these sessions had at least one monkey-only cell recorded simultaneously in the same session.

Since the behavior of the monkey and the human agent was the same during the simultaneous recording of the monkey-only and human only cells within the same session, it is unlikely that possible session-dependent differences in the monkey’s or in the human’s behavior could explain the presence of different categories of cells.

**Figure 5:**
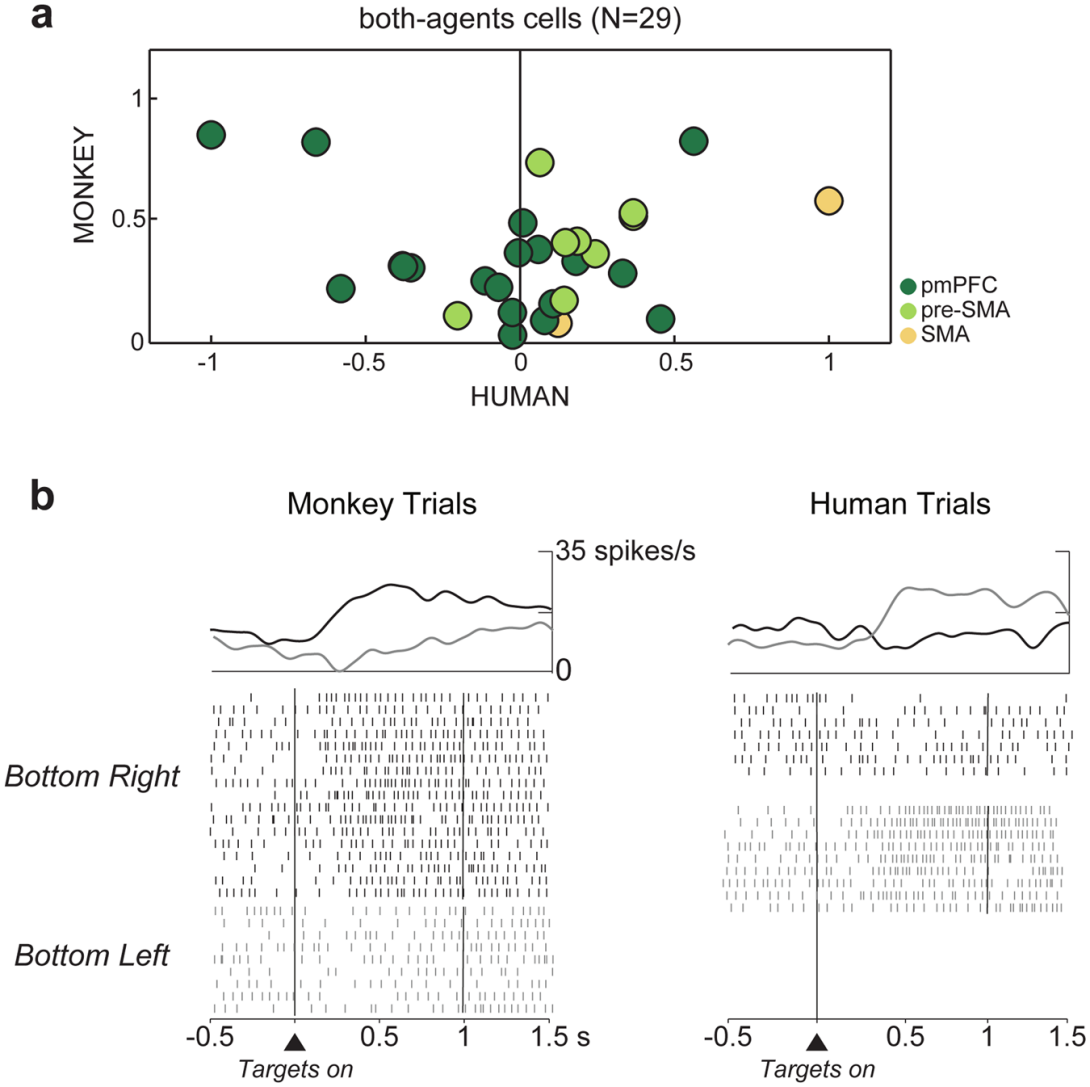
Both-agents cells. (**a**) Scatter plot of the spatial modulation indices in monkey and human trials for both-agents cells. The indexes calculated in the human and in the monkey trials are shown in the abscissa and ordinate, respectively. Cells in the right panel all show congruent preferences. Those on the left have incongruent preferences and were all recorded in pmPFC except one recorded in pre-SMA. (**b**) Both-agents cell with change of spatial preference depending on the actor’s identity. The preferred (bottom right) and antipreferred (bottom left) target positions in monkey trials differ from the preferred (bottom left) and antipreferred (bottom right) target positions in human trials. The cell activity is aligned to targets onset, with the spike density averages above each raster.

The both-agents cells could be further distinguished as congruent cells, when they shared the same preference for target position in both trial types, human or monkey, or incongruent, when they did not. To determine whether the both-agents cells shared the same positional preference in monkey and human trials, the activity in the delay period associated with each of the four target positions in monkey trials was ranked from the most to the least preferred location based on the mean firing rate. The most and least preferred target positions were defined as ‘preferred’ and ‘anti-preferred’, respectively. The position ranks calculated on the activity on the four target positions in monkey trials were then assigned to the human trials. We calculated a spatial modulation index for monkey and human trials separately, using the mean firing rates of the trials corresponding to the preferred and anti-preferred target positions. The calculation was performed using the following formula:

$${\rm{Spatial}}\,{\rm{modulation}}\,{\rm{index}}=({A}_{1}\,-\,{A}_{2})/({A}_{1}+{A}_{2})$$

where *A*_1_ represents the mean firing rate of the preferred target position, and *A*_2_ the mean firing rate of the anti-preferred target position. The scatter plot in Fig. 5a shows, for each brain area, on the abscissa the spatial modulation index calculated for the human trials and on the ordinate the same index calculated for monkey trials. Of 29 both-agents cells in the medial frontal cortex, 17 cells (17/29, 59%) showed congruent preferences, with higher activity for the preferred target position as defined in the monkey trials than for the anti-preferred target position. The remaining cells (12/29, 41%) showed incongruent preferences, that is a change of positional preferences (Fig. 5b). All cells showing incongruent preferences between monkey and human trials were recorded in pmPFC, with the exception of one cell from pre-SMA (Fig. 5a, left panel).

### Population Activity

**Figure 6:**
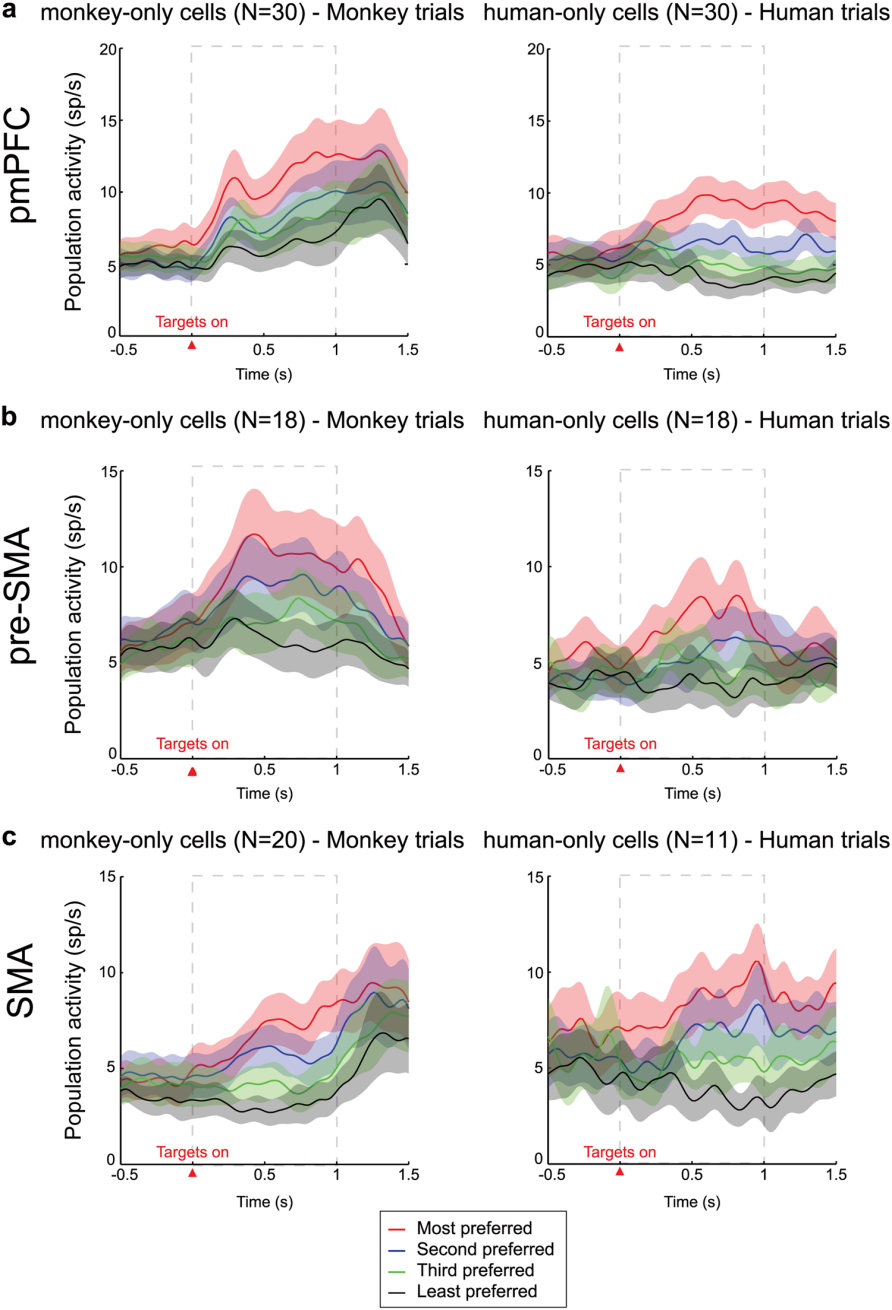
Population histograms. Average population activity of the monkey-only cells (left panels) and human-only cells (right panels) ranked from the most to the least preferred target positions, as determined for pmPFC (**a**), pre-SMA (**b**) and SMA (**c**) cells. The activity is aligned to the targets onset (red triangle). The dashed rectangle indicates the analyzed delay period.

We performed population analyses of monkey-only and human-only cells to examine the time course of the overall selectivity of all cells, for each area. Figure 6 shows the population activity ranked from the most to the least preferred target positions in monkey and human trials, for the monkey-only and the human-only cells, respectively. For both groups of cells of pmPFC and SMA, the activity differences between target positions developed soon after targets appearance, persisting through the delay period and over (Fig. 6a and c) while for the pre-SMA cells the signal started to decrease before the end of the delay period (Fig. 6b).

**Figure 7:**
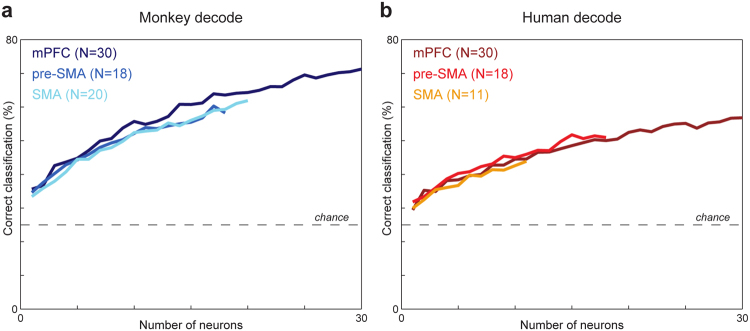
Neuron dropping curves. Each curve represents the percentage of correct single-trial estimations of target position as a function of the number of cells in each group of monkey-only cells (**a**) and human-only cells (**b**) in the delay period for monkey and human trials, respectively, for each of the three brain areas of the medial frontal cortex examined (pmPFC, pre-SMA and SMA). Dashed horizontal lines show the chance level of estimation.

Figure 7 shows the percentage of correct estimations of the target position, in the form of curves derived from neuron-dropping analysis in the delay period (see Materials and Methods), considering monkey trials for the monkey-only cells (Fig. 7a) and human trials for the human-only cells (Fig. 7b), for each area. As expected, estimations of the correct target position were significantly above chance level in all cases, starting from one neuron and increasing as the number of neurons increased. However, we observed a higher proportion of correct target position estimations for the monkey-only than for the human-only cells.

### Previous trial

In addition to the delay period, we also examined the monitoring activity of the previous trial events in the central stimulus period from 0.08 s to 1 s testing whether the neurons were modulated by the previously chosen target and by the agent who carried out the previous trial. Even if the proportion of cells modulated by the previous target position was small, it was almost comparable between areas (Supplementary Fig. S2a). As for the delay epoch, the *post-hoc* analysis on the total cell population determined the proportion of monkey-only, human-only, both-agents, and not significant cells for each recording area (Supplementary Fig. S2b). The proportion of both-agents cells over the total recorded cells was different across areas (χ^2^ (2, *N* = 273) = 8.03, *p* = 0.02), and they were almost lacking in pmPFC. The proportion of monkey-only cells and human-only cells did not differ across areas (monkey-only: χ^2^ (2, *N* = 273) = 4.11, *p* = 0.13; human-only: χ^2^ (2, *N* = 273) = 0.12, *p* = 0.94).

We combined the monkey-only cells and the human-only cells across areas to examine the time course of their selectivity (Supplementary Fig. 2c). The population activity was ranked from the most to the least preferred previous target position in monkey and human trials, separately for the monkey-only and the human-only cells. For both monkey-only and human-only cells, the difference between the ranked target positions before the targets onset (*F*(3, 184) = 2.86, *p* = 0.04 and *F*(3, 160) = 3.87, *p* = 0.01) disappeared soon after the beginning of the delay period (*F*(3, 184) = 0.61, *p* = 0.61 and *F*(3, 160) = 0.76, *p* = 0.52), when the information about the previous target position lost its relevance.

## Discussion

In the present study, we showed that the activity of neurons in pmPFC reflected fundamental aspects of social interaction related to the prediction of others’ future actions.

Predicting or anticipating others’ future actions could be considered an aspect of mentalizing ability. Mentalizing is defined as the ability to reflect, or infer, the mental states of one’s self and other people. It has been associated to the fronto-temporo-parietal network, but it is still debated whether this network could be subdivided in two systems subserving either perceptual or inferential computations^20,21^.

It has long been hypothesized that deficits in social cognition and mentalizing characterize schizophrenia, in which self-other distinction impairments are common^22^, and autism spectrum disorders (ASD). It seems that abnormalities^23,24^ or dysfunctions^25,26^ of medial prefrontal cortex are associated to these disorders. Transcranial magnetic stimulation over dorsomedial prefrontal cortex impairs the ability to distinguish between one’s own and another’s perspective^27^.

To date, the mechanisms at the single cell level underlying the representation of the future behavior of others in Brodmann areas 8 and 9 of the posterior part of dorsomedial prefrontal cortex (pmPFC), a node of the mentalizing system, have not been yet investigated. Although it is not clear whether macaques have a theory of mind, they are at least able to represent others’ knowledge and intentions^1^ that can be investigated. To this aim we used a task in which the monkey switched actor and observer roles with a human agent in different trials. Even as observer, the monkey was required to monitor the other agent’s choices for succeeding when he became the actor in the next trial. This requisite went beyond the demand of observation required for previous paradigms^8,10,28^, and might have played a key role for generating the prediction of others’ choices.

The necessity of monitoring self and others’ choices was a consequence of the task rule to select a non-match to the previously chosen target, irrespective of who completed the previous trial. Importantly, using multiple target positions prevented the prediction of the next target position before its presentation.

Introducing a delay period between the appearance of the targets and the go signal for the action guaranteed a temporal window where it was possible to distinguish target prediction neural signals from action observation activity. For this reason, in this work we focused the analyses on the delay period.

We found neurons coding the target specifically for one agent in the delay period. Human-only cells represented specifically the human agent’s future choice when the monkey was the observer. The presence of this category of cells indicates that a separate population of neurons intervenes to represent the future behavior of another interacting agent rather than relying simply on a simulative activity performed by the same neurons involved in self-made choices. This could be a consequence of the monkeys having not oriented on their own internal simulative world, because the interaction involved another living agent. Future studies should determine whether such neurons would still be active when actors don’t meet face to face and the monkey interacts with an inanimate agent, such as a cursor^29^.

Conversely, monkey-only cells encoded the monkey’s target when he was the actor, but not the other agent’s future target in the human trials. While the monkey-only cells represented the monkey’s future target choice, human-only cells activity represented the future human’s choice in a predictive way. The properties of these two categories of cells were the same as those described for lateral prefrontal cortex (lPFC) neurons^30^. These selective responses revealed separation in the neural representation of self-other behaviors, indicating that the representation of other agents’ behavior did not share a neural substrate with the representation of self-actions.

In addition to these cells, however, we found neurons belonging to a third category: the both-agents cells, which represented the target position or the future action in both monkey and human trials. Within this category of cells, we found neurons sharing the same target preference and others that did not (examined later in the Discussion).

Neurons with shared spatial preferences have been described in motor cortex^31^ and in dorsal premotor cortex^29^ in a task in which the monkey either prepared for an action or for the displacement of an unseen party’s choice toward a target. They found that an overwhelming high proportion of neurons coding for the monkey’s action presented similar activity under the passive condition. Consequently, they interpreted this activity as simulative.

In pmPFC we described a new class of cells: neurons with different spatial preferences depending on the identity of the actor. These neurons changed preferred and anti-preferred target locations, e.g. with a preference for the right target in monkey trials and for the left target (anti-preferred target position in monkey trials) in human trials (Fig. 5b). These neurons appeared to play a different role in human trials, which can be interpreted as an independent predictive activity rather than a simulative one. The presence of such a high proportion of incongruent neurons questions whether the neurons with a shared preference actually represented a simulative activity in pmPFC. One possibility is that the same neurons could flexibly participate in different computations depending on who was acting, shifting to a separate predictive activity when another individual was performing the actions.

We have described a similar switch of selectivity in a non-social context in the lPFC^32^: activity of neurons recorded in both a duration discrimination task and a match-to-sample task, with a similar task structure, switched preferences for which stimulus, the first or the second, was the longest between tasks. As for the previous study, the presence of changes in coding schemes shows the coding flexibility of prefrontal cortex^33^.

The presence of these cells might indicate that the same neural “machinery” used for planning one’s own actions is not necessarily reused as an overarching principle of how the brain works when representing others’ behavior. In our task, the prediction of the other agent’s behavior is based on the very direct knowledge of what others should do based on a given task rule rather than on inferences about others’ perspective, needs, and beliefs. It appears that these cells operate differently from mirror neurons, at least in the sense of not maintaining the same representational coding scheme; this would allow more flexible operation in self- and others-related computations. Changing coding schemes in the pmPFC neurons could also subserve an inhibitor role in human trials, in which the inhibition of self-perspective could be necessary for inferring someone else’s intentions. Future studies should address this possibility with new task designs in pmPFC where these neurons have been found.

Like pmPFC, pre-SMA contained a high proportion of both-agents cells, while in SMA we observed a much smaller proportion of these cells. Although both pre-SMA and pmPFC had both-agents cells, we found incongruent cells only in pmPFC, with the exception of one neuron in pre-SMA. The absence of target coding changes in the pre-SMA neurons could point to a difference between the two areas, suggesting a higher flexibility of the prefrontal neurons.

Our results agree with a previous study in the ACC^34^. The authors showed that ACC cells predicted the cooperative intentions of another individual. However, they did not describe cells lacking overlapping representations for self and others’ behavior as we found in pmPFC.

Moreover, our results in the premotor cortex areas extend the understanding of the role of pre-SMA and SMA in self-other and action differentiation to a predictive role in a period preceding any explicit action; this is in line with what has been found in patients^21^, but in contrast to the lack of activation of these premotor areas reported by neuroimaging studies, which nevertheless reported dorsomedial prefrontal cortex activations^35–38^. It is possible that the lack of activation often reported by neuroimaging studies for premotor areas reflects the averaging out of the activity of neurons with contrasting patterns of activation.

In addition, we found neurons in pmPFC that encoded who the actor was (the monkey, or the human partner), similar to those we previously described in lPFC^30^; these neurons could provide the decoupling of self and others required for social coordination. The presence of this type of neurons in pre-SMA, as well as SMA, was a confirmation of what has been reported previously^2^. Similarly, Simone *et al*.^39^ have found in the ventrolateral prefrontal cortex neurons specifically modulated during the observation of videos in which an agent executed goal directed actions. The self-other distinction should be important, because in a social context it is not only useful to monitor ‘what’ others are doing but also ‘who’ is doing what. The importance of differentiating self from others is also evident from its impairment observed in the compulsory imitative behavior that follows frontal lobe damage^40^.

The importance of agent cells for social interaction has been also suggested by their absence in pre-SMA and cingulate cortex by Yoshida *et al*.^41^ in one monkey with autistic behavior, in whose genes they identified rare coding variants linked to human neuropsychiatric disorders.

Finding cells representing other agents’ choices in pmPFC, and medial premotor areas, is consistent with the pattern of connectivity of pmPFC, which has a role in providing the premotor cortices with information from rostral prefrontal areas^42^.

In addition to a predictive signal in medial frontal cortex, we identified cells involved in self-other related source memory that monitored ‘who’ performed the previous choice and ‘what’ choice was made. This result could shed light on why individuals with autism, in which the functionality of the medial prefrontal cortex is affected^42^, might present with an impairment in the ability to recollect whether they themselves, or someone else, performed a specific action^43,44^.

In summary, we found neurons with changes in coding scheme in pmPFC together with another class of human-only medial frontal neurons common to other two areas, the pre-SMA and SMA. All these neurons might underlie the process of anticipation or prediction of other’s choices beyond a simulation mechanism and could represent a neural substrate of the mentalizing functions.

## Materials and Methods

### Animals

Animal care, housing and experimental procedures conformed to the European (Directive 210/63/EU) and Italian (DD.LL. 116/92 and 26/14) laws on the use of non-human primates in scientific research. The research protocol was approved by the Italian Health Ministry (Central Direction for the Veterinary Service). The housing conditions and the experimental procedures were in accordance with the European law on humane care and use of laboratory animals. Two male rhesus monkeys (*Macaca mulatta*) participated in this study, monkey 1 (10 years old, 9 kg) and monkey 2 (7 years old, 8 kg).

### Task

The monkeys sat in a primate chair, with the head fixed, facing a video touch screen (Microtouch, 19 inches, 800 × 600 pixel resolution) 20 cm away. Figure 1a shows the sequence of events in the Nonmatch-to-goal (NMTG) task. The trial started when a white 7° circle, the central stimulus, appeared at the center of the screen. The monkey had to touch the central stimulus within 2 s, otherwise the trial was aborted and a new trial started. After the central stimulus was touched (with the left hand for monkey 1 and the right hand for monkey 2), a horizontal grey bar (18° × 10°) appeared on the screen (14° above center). The touch on the central stimulus had to be held for 1 s or 1.5 s after which two targets, represented by identical filled grey rectangles (7.1° × 7.7°), appeared in two of four possible screen positions: center left (23.5° left of center), bottom left (17.5° below and 23.5° left of center), center right (23.5° right of center) and bottom right (17.5° below and 23.5° right of center). The disappearance of the horizontal bar, after a delay period of 0 or 1 s from the targets onset, was the go signal. The 0 s delay occurred only in around 9% of trials. A reaching movement to one of the two spatial targets could be done within a 3 s limit. The touch on the chosen target was maintained for a holding-target period of 0.8 or 1.2 s and then a visual feedback was presented around the chosen target for a period of 0.8 or 1.2 s, during which the monkeys continued to touch the target. We used four types of feedback, which, in any given block of trials, differed in shape or color. Two feedback stimuli (a 19.3° × 15.9° blue triangle and a 16.7° empty white circle) signaled an incorrect choice and two others (a 19.3° × 15.9° red triangle and an empty 14.8° × 14.3° white rectangle) signaled a correct choice. Positive and negative feedback stimuli were paired in blocks of trials: the blue and red triangles were always presented in the same block, and the white circle and rectangle were presented in other blocks (Fig. 1a, top right). The two pairs of feedback signals alternated in blocks of 21 correct trials. After the feedback period, on the correctly performed trials, a liquid reward was delivered. Each trial was followed by a period of 1–1.5 s intertrial interval, during which the video screen was black.

On each trial, the previously chosen target reappeared on the screen in the same position together with another target that could appear in one of the other three remaining possible positions, pseudorandomly selected. The new target position could also be the same of that not chosen in the previous trial. The task rule was to reject the previously chosen target position and choose the alternative one. Choosing the same target position that was selected in the previous trial was an error that did not lead to reward delivery, and a correction trial followed. Correction trials consisted of the presentation of the same two positional targets presented in the immediately preceding and incorrectly performed trial. An error in a correction trial was followed by another correction trial. The first choice of every session was always accepted as correct, and the reward was delivered for any chosen target. It is important to emphasize that our NMTG task cannot be compared to a classical alternation task because it involves more than two target positions. This aspect of the task allowed us to study specifically the neural activity modulation to the previous chosen target position in the central stimulus period, when the future target position cannot be determined yet.

### Interaction with Human Partner

During the recording sessions (45 for monkey 1 and 75 for monkey 2) monkeys interacted with a human partner. Figure 1b shows an example sequence of trials. The human partner was sitting on the monkey’s right side, close to the animal. The human partner could start his turn as actor only after the monkey completed a trial, indicating his turn by moving his hand toward the center of the screen only during the intertrial period, when the screen was totally black, ready to touch the central stimulus when it appeared. No other external cues signaled the turn of each agent at the beginning of each trial. It was the human agent who decided the turns. After the human partner moved his hand toward the screen, monkeys were trained to let human partners perform a sequence of trials (from 1 to 4) without interfering. For example, Fig. 1b shows the human partner performing two consecutive trials. The human partner never interfered while monkey was executing a trial, and vice versa. When the human partner drew back his arm at the end of a sequence of trials, that was the signal the monkeys used to approach to the central stimulus for starting a new trial.

The interaction with the human started only after monkeys had learned the task alone. The human partner could be one of two persons during the recording sessions.

Using a human as a partner reduced the possibility to have in the observing monkey a predictive behavior based on not controlled and not correctable stereotyped behaviors or on anticipatory arm or ocular motor signals from another monkey as a partner. The position of the human on the monkey’s side rather than in front also prevented access to the human’s eye movements.

Moreover, the human partner could reliably make only correct choices, so that the reward was always delivered to the monkey, both in monkey and human trials.

During the task, the eye position of monkeys was recorded but not placed under experimental control. Unfortunately, the eye data from monkey 2 could not be examined because of a technical problem in the eye data recordings. The eye data from monkey 1 was examined only for sessions without a problem in the eye data recordings (32/45 sessions).

### Trial Types

During the monkey-human interaction phase of the experiment we assigned monkey trials to two categories: *not interactive* and *interactive* trials, the latter designed to test the monkeys’ ability to monitor the past trial performed by the human agent. The *not interactive* trials were the trials performed by the monkeys after a trial performed by the monkeys themselves. The *interactive* trials were the trials performed by the monkey preceded by a trial performed by the human partner (Fig. 1b). The *interactive* trials could be performed correctly only by monitoring the human choices.

We referred to these *not-interactive* and *interactive* trial classifications only for the behavioral analyses. For the neural analyses, we referred to *monkey* or *human* trials based on which agent was performing the trial.

### Surgical techniques

During the training period, a head holding device was implanted under aseptic surgical conditions. The animals were anesthetized with isoflurane (Abbott Laboratories) through a constant flux of isoflurane/O2 mixture (1–3%, to effect). Antibiotics and analgesics were administered postoperatively. Before the recording started, again under general anesthesia, a recording cylinder (18 mm in diameter) was implanted stereotaxically.

The chamber provided access to three areas of medial frontal cortex: pmPFC, pre-SMA, SMA. Recording sites were localized relative to the principal sulcus and the arcuate sulcus after opening the dura matter for a different experimental protocol involving the same animal in monkey 2, and based on stereotaxic coordinates in monkey 1. Anatomical boundaries are illustrated on a schematic representation of the brain of monkey 2 in Fig. 4b, top left. The posterior border of pmPFC is defined as in Yoshida *et al*.^41^ and the border between pre-SMA and SMA is based on the anatomical work of Rizzolatti *et al*.^45^.

### Data collection

To control stimulus presentation and reward delivery, and to record touches on the screen and categorize the trials, we used a non-commercial software package, CORTEX (http://www.nimh.nih.gov/labs-at-nimh/research-areas/clinics-and-labs/ln/shn/software-projects.shtml). We monitored the eye position through the ViewPoint Eye Tracker system (Arrington Research, Scottsdale, USA). Neural activity of single units was recorded extracellularly with a five-channel multi-electrode system (Thomas Recording, Giessen, Germany). Electrodes were quartz-insulated platinum-tungsten fibers (80-μm diameter, 0.8- to 2.5-MΩ impedance) and were inserted transdurally with microdrives (Thomas Recording). Electrical signals were amplified and filtered, and single units were isolated online with a TDT system (Tucker-Davis Technologies, TDT, Alachua, USA). The same TDT system was used to record the eye movements. The data analyses were performed using MATOFF software^46^.

### Data Analysis

#### Behavior

For each monkey, we analyzed the human performance, the proportion of executed and consecutive trials, as well as the number of monkey trials after which the human partner initiated a new sequence of trials. The percentage of correct trials and the reaction time (RT) of each monkey was calculated for *not interactive* and *interactive* conditions separately.

#### Single neuron activity

We recorded non-simultaneous neural activity from three areas of medial frontal cortex: pmPFC, pre-SMA and SMA. All neurophysiological analyses were performed separately for each area. We evaluated the activity of neurons in correct trials preceded by a correct trial. We selected only current trials with a 1 s delay.

We analyzed the neural activity during two periods of the task. The central stimulus period, extended from 0.08 s to 1 s after the touch started, was used to assess whether the activity in the current trial changed depending on the agent and on the target chosen in the previous trial.

The delay period, extended from 0.08 s to 1 s after the appearance of the peripheral targets, was used to assess whether the activity in the current trial changed depending on the agent and the target chosen in the current trial.

All recorded neurons were not preselected based on task relationship assessment.

We performed two, separate, 2-way ANOVAs for each period.

For the delay period, the agent performing the trials and the current chosen target position were used as factors. For the central stimulus period, the previous agent and the previous chosen target position were used as factors. For both periods, the *post-hoc* testing (Fisher’s least significance difference, *p* < 0.05) was repeated on the same pool of cells used for the 2-way ANOVA analysis to assess whether there was at least one difference in activity between the four target positions, separately in monkey and in human trials. We did not consider any further positional difference across agents.

With this analysis, we identified 3 categories of cells: 1) cells showing at least one positional difference between the four target positions only in monkey trials; 2) cells showing at least one positional difference between the four target positions only in human trials; 3) cells responding differently to the four target positions both in monkey and in human trials. For simplicity, we called each category as: 1) monkey-only, 2) human-only, and 3) both-agents cells. All the other cells with no significant positional differences either in monkey or in human trials were discarded from further analysis.

The both-agents cells were further analyzed (described in detail in Results) to identify whether the differences between the four target positions in monkey and human trials were shared or not.

#### Population Activity

The two categories of only-monkey and human-only cells identified from the *post-hoc* analyses were treated separately for population activity analysis.

For each cell of each category the neural activity for the four correct target positions was ranked from the most to the least preferred location, according to the highest and lowest mean firing rate in each analyzed period.

The signals ranked in the delay period were plotted in the form of population histograms in steps of 20 ms from 0.5 s preceding to 1.5 s following the targets appearance for showing the cells’ modulation for the current chosen target in the current trial.

The signals ranked in the central stimulus period were plotted in steps of 20 ms from 1.5 s preceding to 1.5 s following the targets appearance for showing the modulation for the previous chosen target in the current trial. A one-way ANOVA established the differences between ranked target positions 1 s before and 1 s after the targets appearance. The *post-hoc* testing (Fisher’s least significance difference, *p* < 0.05) determined which ranked positions were different for each period, separately.

#### Neuron-dropping analysis

To assess the correct estimation of the target position in monkey and human trials, neuron-dropping curves were computed for each neuron of monkey-only and human-only cells, separately, in the delay period. The computation started from one neuron randomly selected from each cells’ category. For the selected neuron and for a given condition, one test trial corresponding to one correct target position among the 4 possible positions was randomly chosen and temporarily removed. The mean firing rates of all remaining trials were assembled in a look-up table. The look-up table was a matrix of mean firing rates for the 4 target positions. The difference in firing rate between the test trial and the average rate for each remaining trial type was calculated and then ranked between 1 and 4, 1 indicating the closest match. The selected test trial was then classified as being of the trial type with the smallest sum of ranks across the selected neurons. If the classified trial type and the test trial type shared the same location, then the classification of the target position was correct. We computed neuron-dropping curves for one neuron to the total number of neurons, considering different ensembles for the population estimation.

This operation was repeated 1000 times, for each of the 4 conditions, to determine how often this computation led to a correct estimation of the target position, separately for monkey and human trials.

### Data availability

The datasets generated and/or analyzed during the current study are available from the corresponding author on reasonable request.

## Electronic supplementary material

Suppplementary materials
