# Supplementary material for: Neural activity in macaque medial frontal cortex represents others’ choices
Source: Sci Rep. 2017 Oct 4;7:12663. doi: 10.1038/s41598-017-12822-5 (PMC5627293; doi:10.1038/s41598-017-12822-5)
Supplement: Supplementary file 1 — Suppplementary materials [file 41598_2017_12822_MOESM1_ESM.pdf]

Title: Neural activity in macaque medial frontal cortex represents others' choices.

Rossella Falcone[1], Rossella Cirillo[1], Stefano Ferraina[1], Aldo Genovesio[1*].

Affiliation:

[1] Department of Physiology and Pharmacology, Sapienza - University of Rome, Piazzale Aldo Moro 5, 00185 Rome, Italy.

[*]Correspondence: aldo.genovesio@uniroma1.it

Number of Pages: 4

Number of Figures: 2

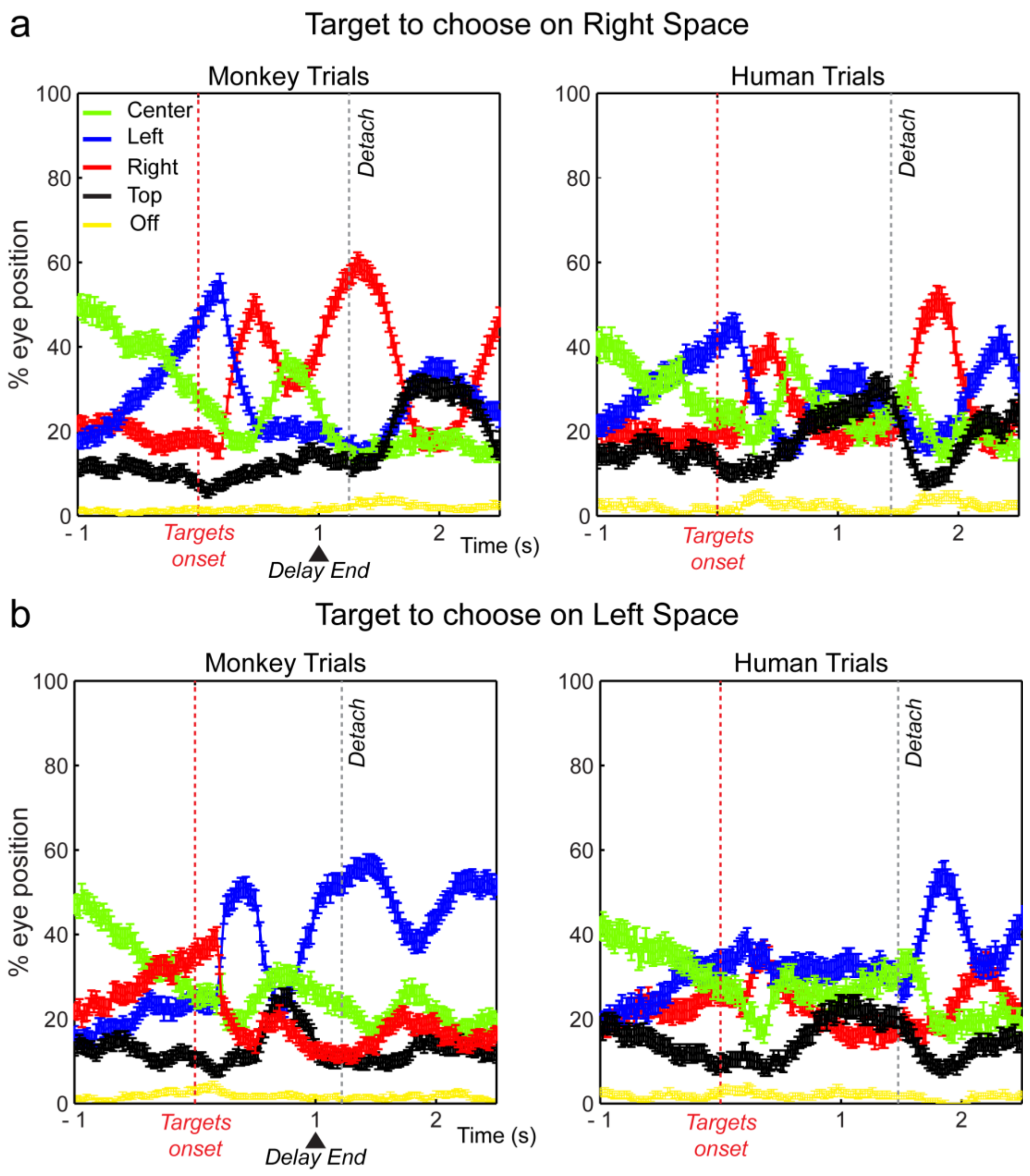

Figure S1. Oculomotor Behavior.

Percentage of times in which the eye of monkey 1 was on the right (red curves), left (blue curves), center (green curves), on the top space of the screen (black curves), and off-screen (yellow curves). Each curve represents the percentage of the eye position computed in steps of 20 ms bins starting in the last 1 s of holding central stimulus period before the targets onset (dashed red line) to 1.5 s after 1s of delay. The percentages were calculated separately for correct monkey and human trials, with delay 1s, preceded by a correct trial and where the two

targets (one to choose and the other one to discard) appeared on the opposite spaces of right and left. (**a**) Percentage of times in which the eye was on the screen and off-screen when the target to choose was on the right space either when monkey (left panel) or human (right panel) were the actors. (**b**) Percentage of times in which the eye was on the screen and off-screen when the target to choose was on the left space either when monkey (left panel) or human (right panel) were the actors (data are represented as mean ± SEM).

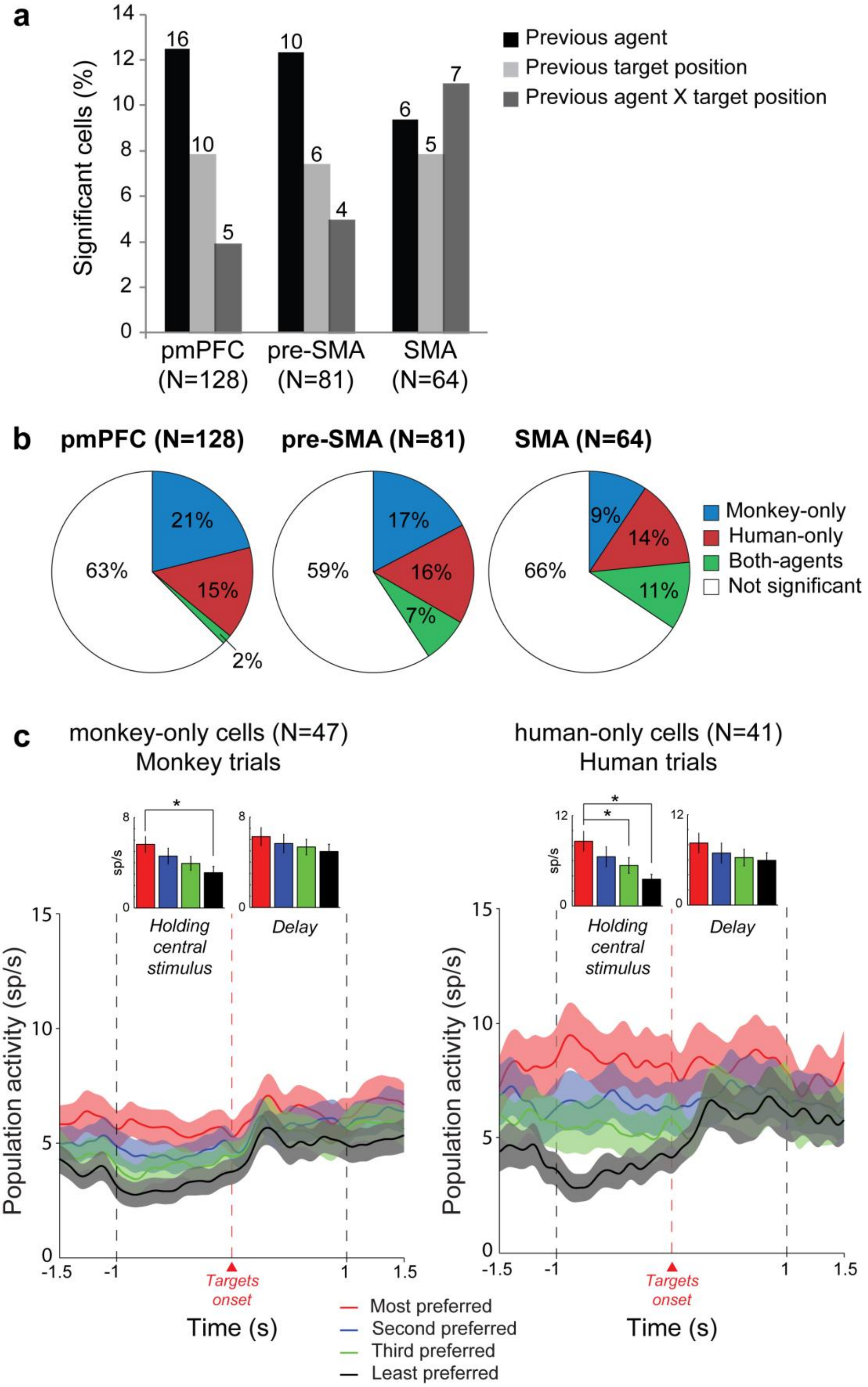

a
Significant cells (%)
14
12
10
8
6
4
2
0
16
10
5
10
6
4
6
5
7
pmPFC (N=128)
pre-SMA (N=81)
SMA (N=64)
Previous agent
Previous target position
Previous agent X target position
b
pmPFC (N=128)
pre-SMA (N=81)
SMA (N=64)
21%
15%
2%
63%
17%
16%
7%
59%
9%
14%
11%
66%
Monkey-only
Human-only
Both-agents
Not significant
c
monkey-only cells (N=47)
Monkey trials
human-only cells (N=41)
Human trials
sp/s
Holding central stimulus
Delay
Population activity (sp/s)
Targets onset
Time (s)
-1.5
-1
1
1.5
Most preferred
Second preferred
Third preferred
Least preferred

Figure S2. Previous Trials.

(a) Percentages of cells with a significant effect of previous agent, previous target position and their interaction during the central stimulus period for each of the three brain areas: pmPFC, pre-SMA and SMA.

(b) Percentages of cells with a different response according to the previous target position only in monkey trials (monkey-only cells), only in human trials (human-only cells), or in both monkey and human trials (both-agents cells) and with no different modulation of the activity according to the previous target position (not significant cells) for each brain area of the medial frontal cortex (pmPFC, pre-SMA and SMA).

(c) Average population activity of the monkey-only (on the left panel) and human-only (on the right panel) cells ranked from the most to the least preferred previous target positions as determined in the monkey trials (on the left) and human trials (on the right). The activity is aligned to the targets onset (red triangle). The dashed lines indicate the two periods of 1 s each, before and after the targets onset. For each period, the mean firing rates of each ranked position were calculated (see inset bar graphs – data are represented as mean ± SEM). Post-hoc comparison (Fisher's LSD, $^{*}p < 0.05$) determined which ranks were different.
